# Phase I study of vemurafenib in children with recurrent or progressive BRAF^V600E^ mutant brain tumors: Pacific Pediatric Neuro-Oncology Consortium study (PNOC-002)

**DOI:** 10.18632/oncotarget.27600

**Published:** 2020-05-26

**Authors:** Theodore Nicolaides, Kellie J. Nazemi, John Crawford, Lindsay Kilburn, Jane Minturn, Amar Gajjar, Karen Gauvain, Sarah Leary, Girish Dhall, Mariam Aboian, Giles Robinson, Janel Long-Boyle, Hechuan Wang, Annette M. Molinaro, Sabine Mueller, Michael Prados

**Affiliations:** ^1^ Department of Pediatrics, NYU Langone Health, New York, NY, USA; ^2^ Doernbecher Children’s Hospital, Oregon Health and Science University, Portland, OR, USA; ^3^ Rady Children’s Hospital, San Diego, CA, USA; ^4^ Center for Cancer and Blood Disorders, Brain Tumor Institute, Children’s National Health System, Washington, D.C., USA; ^5^ Department of Pediatrics, Perelman School of Medicine, University of Pennsylvania, Philadelphia, PA, USA; ^6^ St. Jude Children’s Research Hospital, Memphis, TN, USA; ^7^ Washington University, St Louis, MO, USA; ^8^ Fred Hutchinson Cancer Research Center, Seattle, WA, USA; ^9^ University of Alabama Division of Hematology and Oncology, Birmingham, AL, USA; ^10^ Department of Radiology, Yale University, New Haven, CT, USA; ^11^ Department of Pharmacology, University of California San Francisco, San Francisco, CA, USA; ^12^ Center for Translational Medicine, School of Pharmacy, University of Maryland, Baltimore, MD, USA; ^13^ Department of Neurological Surgery, University of California San Francisco, San Francisco, CA, USA; ^14^ Department of Neurology, University of California San Francisco, San Francisco, CA, USA; ^15^ Department of Pediatrics, University of California San Francisco, San Francisco, CA, USA; ^16^ University Children’s Hospital Zürich, Zürich, Switzerland; ^*^ Co-Senior authors

**Keywords:** BRAF^V600E^, pediatric glioma, vemurafenib, clinical trial

## Abstract

**Background:** BRAF^V600E^ mutation is present in a subset of pediatric brain tumors. Vemurafenib is an oral, selective ATP-competitive inhibitor of BRAF^V600E^ kinase. The goal of this multi-center study conducted through the Pacific Pediatric Neuro-Oncology Consortium (PNOC) was to determine the recommended phase 2 dose (RP2D) and dose limiting toxicities (DLTs) in children < 18 years with recurrent or progressive BRAF^V600E^ mutant brain tumors.

**Results:** Nineteen eligible patients were enrolled. Eleven patients had received three or more prior therapies. Data reported are from the start of treatment for the first patient (April 30 2014) through August 31 2019. The RP2D was defined as 550 mg/m^2^ twice daily after DLT criteria adjustment for rash. Related grade ≥ 3 adverse events included secondary keratoacanthoma (*n* = 1); rash (*n* =16); and fever (*n* = 5). Subjects received a median of 23 cycles (range 3–63). Four patients remain on treatment. Centrally reviewed best radiographic responses included 1 complete response, 5 partial responses, and 13 stable disease. The steady-state area under the curve (AUC_0-∞_median) was 604 mg*h/L (range 329–1052).

**Methods:** Vemurafenib was given starting at 550 mg/m^2^, twice daily which corresponds to the adult RP2D. Adverse events were graded using the NIH Common Terminology Criteria for Adverse Events (CTCAE) version 4.0. Central imaging review was performed. Pharmacokinetic sampling was performed.

**Conclusions:** Vemurafenib has promising anti-tumor activity in recurrent *BRAF* V600E-positive brain tumors with manageable toxicity. A phase 2 study is ongoing (NCT01748149).

## INTRODUCTION

BRAF^V600E^ is one of the most common oncogenic mutations in human tumors, found in 50% of metastatic melanomas, 10% of metastatic colon carcinomas, and 30% of papillary thyroid carcinomas [1]. This point mutation results in a constitutively active form of BRAF that functions as a monomer and is resistant to feedback inhibition [2]. Small molecule inhibitors that specifically target the BRAF^V600E^ kinase domain have been developed and have shown significant, albeit transient, activity in adult metastatic melanomas with BRAF^V600E^ mutations [3]. An unexpected side effect of this class of inhibitors is a high risk of secondary squamous cell carcinoma (keratoacanthoma). These occur in approximately 25% of treated adults and have been demonstrated to contain activating RAS mutations (or less frequently NOTCH or TGF deletions) and show growth stimulation through paradoxical activation of wild type RAF dimers by BRAF inhibitors [4].

Gliomas are the most common subgroup of pediatric brain tumors [5]. Children with low grade gliomas (WHO grade 1 and 2) have an excellent prognosis when these lesions can be totally resected, but often require adjuvant therapy when gross total resection cannot be achieved. Children with high grade gliomas (WHO grade 3 and 4) have a poor prognosis, despite aggressive multimodal therapy and no standard therapy, other than surgical resection and radiotherapy, has been established [6].

Until recently, adjuvant treatment for children with gliomas was limited to cytotoxic chemotherapy and radiation due to the lack of knowledge of the biological drivers of these tumors and a lack of available agents that could target such drivers [7]. Over the past ten years, many groups have demonstrated a high frequency of alterations in the RAS/RAF/MAPK pathway in pediatric gliomas. BRAF^V600E^ mutations, in particular, are found in 5% of malignant astrocytomas, 9% of pilocytic astrocytomas, 50% of gangliogliomas and 66% of pleomorphic xanthoastrocytomas [8]. Our group demonstrated significant anti-tumor efficacy of PLX4720 (tool compound analog of the BRAF^V600E^-specific inhibitor vemurafenib) in intracranial xenografts harboring BRAF^V600E^-mutant gliomas, while showing no efficacy against BRAF-wild type xenografts [9].

Herein we report on a multi-center phase 1 study conducted through the Pacific Pediatric Neuro-Oncology Consortium (PNOC) of vemurafenib in children < 18 years of age with recurrent or progressive BRAF^V600E^ mutant brain tumors. At the time of trial development, there were no published reports of vemurafenib safety, efficacy, CNS penetration or pharmacokinetics in children with gliomas.

## RESULTS

### Subject characteristics

Among 19 eligible patients, one was not compliant with medications during the DLT period and therefore not fully evaluable for estimation of RP2D or PK analysis. Table 1 summarizes the subject characteristics. The most common histology was pilocytic astrocytoma (n=10). Although this trial was open to both low and high-grade tumors, only patients with low-grade tumors enrolled in the safety study. While this trial was open at multiple sites, the relative scarcity of pediatric patients with recurrent BRAF^V600E^ mutant brain tumors led to expected relatively slow accrual.

**Table 1:** Patient characteristics

**Age in years, at study enrollment, median (min, max)**	9 (3–17)	
**Number of courses of vemurafenib: median (min, max)**	23 (4–64)	
**Number of Prior Therapies**	**Number of patients**	
One	4	
Two	4	
Three or more	11	
	**Number**	**Percentage**
**Gender**		
Males	9	47.4
Females	10	52.6
**Race**		
White, non-Hispanic	14	73.7
Black	2	10.5
Unknown	3	15.8
**Diagnosis**		
Astrocytoma (NOS)	1	5.3
Fibrillary Astrocytoma	1	5.3
Pilocytic Astrocytoma	10	52.6
Ganglioglioma	5	26.3
Pleomorphic Xanthoastrocytoma	2	10.5

### Toxicities

The most common toxicity was maculopapular rash (Table 2). This is a known side effect of vemurafenib and responded well to holding the drug and supportive care. Two of the three patients initially treated at both Dose Level 0 and −1 had grade 3 rash and met DLT criteria. As these rashes resolved shortly after holding drug with appropriate supportive care and did not recur with restarting drug, we subsequently amended the study to exclude rash that resolved within 7 days with supportive care as a DLT. As a consequence, rather than further dose reductions, additional patients were treated at dose level −1. Dose level −1 was subsequently declared safe without further rash and patients were then escalated back up to dose level 0, which was also well tolerated and was determined as the RP2D.

**Table 2:** Number of grade 2 & 3 toxicities probably, possibly or definitely attributable to vemurafenib

Adverse Event	Grade 2	Grade 3
**Alanine aminotransferase increased**	0	1
**Alkaline phosphatase increased**	1	1
**Alopecia**	1	0
**Anorexia**	2	0
**Arthralgia**	3	0
**Aspartate aminotransferase increased**	2	0
**Blood bilirubin increased**	3	1
**Bullous dermatitis**	1	0
**Creatinine increased**	2	0
**Diarrhea**	2	0
**Dry skin**	3	0
**Electrocardiogram QT corrected interval prolonged**	0	1
**Erythema multiforme**	1	0
**Erythroderma**	1	0
**Fatigue**	2	0
**Fever**	1	2
**Gastrointestinal disorders - Acid reflux**	1	0
**Headache**	1	0
**Hypertension**	2	0
**Hypophosphatemia**	1	0
**Investigations - Plantar hyperkeratosis with pain**	1	0
**Lymphocyte count decreased**	2	1
**Mucositis oral**	1	0
**Nausea**	1	0
**Neoplasms benign, malignant and unspecified (incl cysts and polyps) - keratoacanthomas**	0	1
**Palmar-plantar erythrodysesthesia syndrome**	1	0
**Photosensitivity**	0	1
**Pneumonitis**	0	1
**Pruritus**	1	1
**Rash maculo-papular**	12	10
**Skin and subcutaneous tissue disorders – Dermatitis, heat rash, photoonycholysis**	3	0
**Skin infection**	1	0
**Somnolence**	1	0
**Weight loss**	1	0

One thirteen-year-old patient developed several facial lesions during Cycle 4 that were tissue confirmed squamous cell carcinoma (SCC).

### Pharmacokinetics

A total of 19 subjects underwent pharmacokinetic sampling. One subject with poor compliance was removed from the final analysis. The steady-state AUC_0-∞_ median was 604 mg*h/L (range 329–1052 mg*h/L). Pharmacokinetic analysis demonstrated a significant accumulation factor (approximately six-fold) over time with each vemurafenib dose. In an attempt to correlate exposure with response, patients were divided into those having stable disease (SD) versus those having PR or CR. Patients with SD had 11% lower AUCss (586 mg*hr/L) compared to PR+CR (657 mg*hr/L). However, a logistic regression model using AUCs as a predictor of PR+CR was not significant given the observed variability.

### Patient outcomes

**Figure 1:**
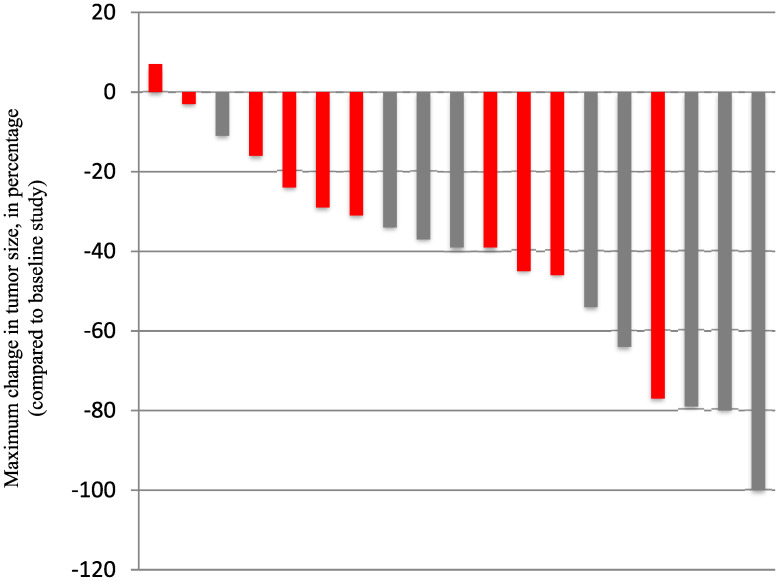
Depicted is the centrally reviewed “best response” per patient, based on the maximum change compared to on study MRI. Each bar represents a patient. Grey bars depict patients treated on dose level 0 and red bars show subjects treated on dose level-1.

**Figure 2:**
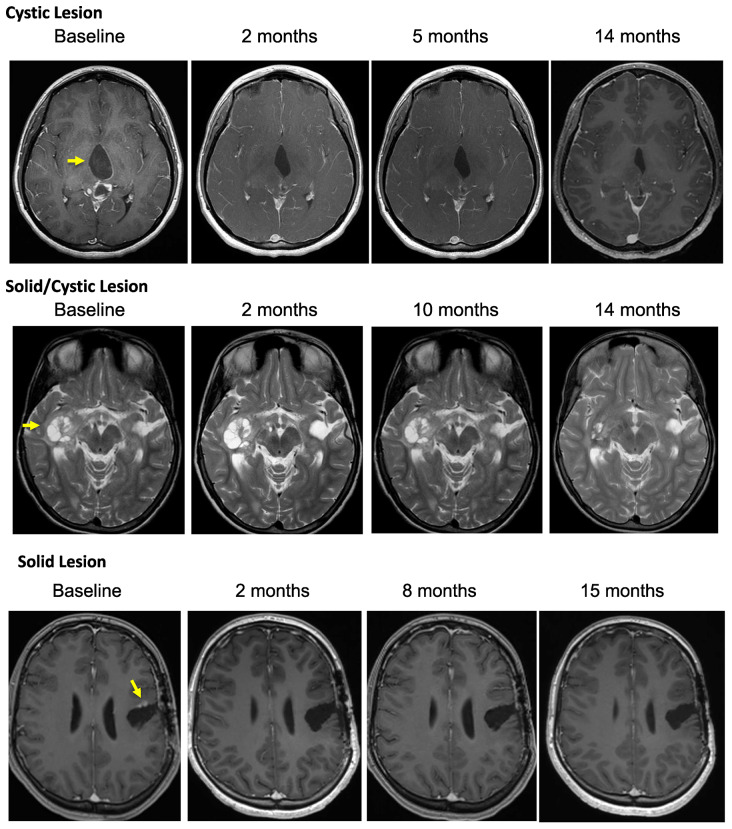
Depicted are representative images of subjects treated on PNOC-002 demonstrating. (**A**) regression of a contrast enhancing cystic lesion on a contrast, T1 weighted MR image over time; (**B**) regression of a solid/cystic lesion on a T2 weighted MR image over time; (**C**) regression of a contrast enhancing solid lesion on a contrast, T1 weighted MR image over time.

Patient outcomes are reported for the 19 patients treated. The median number of vemurafenib courses was 23 (range 3 to 63). Centrally reviewed best radiographic responses included 1 CR, 5 PR, and 13 patients with SD (Figure 1). Solid tumor component was measured for determination of outcomes in 10 patients, with the remaining 9 patients with no measurable solid component undergoing measurement of solid/cystic lesion for outcome determination. Figure 1 demonstrates best response percent decrease in tumor size during the course of treatment with vemurafenib. As shown in Table 3, responses were durable with some patients having continuous response for over 40 months. Cystic lesions appeared to decrease more in size compared to the solid lesions with vemurafenib therapy (Table 4). Interestingly, we found that 10 of the patients in our cohort who presented with contrast-enhancing tumors were found to develop loss of enhancement during treatment. Examples of radiographic responses are shown in Figure 2, with representative solid, cystic, and mixed solid/cystic lesions.

**Table 3:** Patient Outcomes

**Patient ID-Pathology^1^**	**Enrolled Dose (mg/m2)**	**On treatment date**	**Off treatment date^2^**	**Dose Limiting Toxicity**	**Best Response^3^**	**Number of cycles^4^**	**Reason Off Treatment^5^**	**Date of progression**
1 - low grade astrocytoma NOS	550	4/30/2014	5/27/2015	yes	PR	14	Completed Therapy	9/1/2015
3 - pilocytic astrocytoma	550	9/29/2014	9/2/2015	no	SD	12	Completed Therapy	n/a
4 - pilocytic astrocytoma	550	10/16/2014	+	yes	SD	64	n/a	n/a
5 - PXA	420	12/4/2014	8/13/2015	yes	SD	9	Adverse event	n/a
6 - fibrillary astrocytoma	420	1/29/2015	12/28/2015	no	SD	12	Completed Therapy	n/a
7 - pilocytic astrocytoma	420	3/9/2015	5/31/2016	yes	SD	16	Patient decision^*^	n/a
10 - ganglioglioma	420	8/20/2015	9/15/2016	no	SD	14	Disease progression	9/13/2016
11 - pilocytic astrocytoma	420	9/30/2015	2/13/2019	no	SD	44	Patient decision^*^	n/a
12 - ganglioglioma	420	12/8/2015	2/11/2019	no	SD	41	Patient decision^*^	4/19/2019
13 - pilocytic astrocytoma	420	12/31/2015	4/8/2016	no	PR	4	Adverse event	n/a
14- pilocytic astrocytoma	420	4/14/2016	5/17/2018	no	SD	27	Patient decision^*^	n/a
15 -ganglioglioma	420	5/12/2016	+	no	SD	43	n/a	n/a
16 - ganglioglioma	420	6/15/2016	+	no	SD	42	n/a	n/a
17 -pilocytic astrocytoma	550	9/29/2016	+	no	PR	38	n/a	n/a
18 - PXA	550	10/3/2016	8/8/2018	no	CR	24	Patient decision^*^	n/a
19 - pilocytic astrocytoma	550	11/10/2016	8/31/2018	no	SD	24	Patient decision^*^	n/a
20 - pilocytic astrocytoma	550	1/11/2017	10/26/2018	no	PR	23	Adverse event	12/11/2018
21 - pilocytic astrocytoma	550	2/22/2017	4/25/2018	no	PR	15	Adverse event	12/10/2018
22- ganglioglioma	550	2/21/2017	11/8/2018	no	SD	22	Poor compliance	n/a

1. NOS = not otherwise specified; PXA = Pleomorphic Xanthoastrocytoma

2. + Indicates that the patient remains on treatment as of 8/31/2019

3. Best response by imaging. PR = partial response; SD = stable disease

4. Median number of cycles = 23

5. Completion of protocol is defined as completing 12 cycles therapy.

^*^Patient decision to stop treatment with stable disease after 12 or more cycles; treatment was allowed to continue indefinitely as long as no toxicity, progression, or patient preference

**Table 4:** Patients listed by their study numbers and shown are product of bi-dimensional tumor measurements for each tumor lesion. Some patients had more than one lesion

Patient #	Cystic	Solid/Cystic	Solid	Central review Best Response
1		−93	−79	PR
3	−78	−37		SD
4			−11	SD
5		−24		SD
6	−12	7		SD
7	−73		−29	SD
10	−89	−39		SD
11			−45	SD
12		−32	−16	SD
13		−77		PR
14		−3		SD
15			−46	SD
16	−92	−100	−31	SD
17	−100	−80		PR
18			−100	CR
19			−34	SD
20		−82	−54	PR
21	−72	−64		PR
22		−39		SD

Of the 19 patients, only one patient (patient 10) progressed on therapy (Table 3). Three patients (patients 1,3, and 6) came off drug because they had completed therapy (originally defined as 12 months). Eleven patients came off drug due to adverse events or patient decision. Four patients remain on therapy. Of the fourteen patients total that came off drug without progressive disease, only four progressed during the 12-month protocol-defined follow up period (Table 3).

## DISCUSSION

The discovery of BRAF^V600E^ mutations in a wide spectrum of gliomas has led to optimism that these tumors can be therapeutically targeted by potent inhibitors of the mutant form of BRAF developed for the treatment of melanoma. Herein, we report that vemurafenib is safe in children with BRAF^V600E^ gliomas and has promising anti-tumor efficacy.

Vemurafenib was approved for the treatment of BRAF-mutant melanoma in 2011. Toxicities in adults with melanoma include rash, arthralgias and secondary malignancies (mostly SCC) [3]. The latter are of particular concern when considering this therapeutic approach for children, particularly for low grade tumors. In this phase 1 study, we found that the adult dose equivalent of 550 mg/m^2^ was well tolerated in children with a similar safety profile seen in adult subjects. These results are similar to the small study of vemurafenib in adolescents with melanoma [10] and the recently reported pediatric experience with Langerhans Cell Histiocytosis [11]. While we encountered rash as a DLT in our initial cohort of patients, this was not seen once we amended our protocol with more universally used criteria for grading transient grade 3 non-hematologic toxicities in patients receiving BRAF inhibitors. Interestingly, one patient developed SCC while on vemurafenib therapy in our study. This patient did not have any concerning lesions at baseline and developed facial SCC during cycle 4 of therapy. Biopsy confirmed the diagnosis and genomic analysis of the SCC revealed TGF-beta receptor homozygous deletions, without any additional mutations affecting the RASA/RAF/MAPK pathway. TGF-beta signaling is known to have a tumor suppressive effect in skin stem cells and loss of TGF-beta signaling has been associated with SCC development in adults treated with BRAF inhibitors [4]. Of note, one of the six adolescents treated with vemurafenib in the melanoma study also developed an SCC [10].

Only one of the 19 patients who participated in this phase 1 study developed progression while on therapy. Four patients remain on therapy with a mean of > 23 cycles of therapy. Our study found a high proportion of objective radiographic responses that were durable, with 1 CR, 5 PRs, and 13 SDs as the centrally-reviewed best responses on therapy. The recently reported phase I/IIa experience with the BRAF^V600E^ inhibitor dabrafenib in children with recurrent pediatric low grade glioma reported 1 CR, 13 PR, 11 SD and two progressive diseases as best responses in a cohort of 32 patients [12, 13]. This is of particular interest as both drugs have been shown to have poor CNS penetration in animal models [14]. It is important to point out that the dabrafenib study used the RANO criteria [15] to measure objective response, while our study used a modified RANO criteria which included tumor cysts in measurements.

One of the pressing issues facing the pediatric neuro-oncology community is the optimal duration of therapy for targeted agents. Of our fourteen patients that halted treatment for either toxicity, patient decision, or completion of therapy, four patients progressed during 12 months of follow up (Table 3). This suggests that while responses can be durable in some patients after halting drug, the ideal duration of therapy remains to be determined. Of note, three of our four patients that developed progressive disease off drug progressed within four months of stopping therapy and so careful early monitoring of patients who stop vemurafenib therapy is warranted.

The first reported trial of a BRAF inhibitor (sorafenib) for pediatric gliomas did not report any secondary skin cancer formation, but instead reported a significant stimulation of glioma tumor growth, which we did not observe in our cohort [16]. The underlying etiology of the sorafenib growth activation was thought to be the presence of the KIAA1549: BRAF fusion protein in the majority of enrolled patients. In contrast, our study restricted eligibility to patients with the BRAF^V600E^ mutation and specifically excluded patients with the KIAA1549: BRAF fusion, NF1, or any other known RAS-opathy. Appropriate subject selection and detailed preclinical characterization will be critical as targeted therapies such as vemurafenib will be integrated in the care of these children.

Pharmacokinetic analyses revealed similar drug exposure and kinetics as in adult patients [17]. It is well recognized there are often differences in pharmacodynamic endpoints between pediatrics and adults. Limitations exist when adult PK data and study endpoints are applied to a pediatric population particularly in early phase trials where likelihood of success is low. Additionally, the historically applied maximum tolerated dose (based on toxicity) is often not equivalent to the maximum efficacious dose (based on biomarkers). Given the lack of data for reliable biomarkers in this rare disease population we specifically designed the study collection of new data (both PK and biomarkers) so that it can be combined with future trials to enhance sample size and better defining exposure-response relationships to optimize the use of this drug in the pediatric population. While there was a correlation between drug exposure and radiographic response, this was not statistically significant in this cohort. Within the phase 2 study that is actively ongoing we will also assess if crushing vemurafenib for liquid formulation leads to similar exposure which is an important assessment when developing therapeutic options for children.

In summary, we report that vemurafenib is tolerable and efficacious in children with recurrent low grade gliomas with *BRAF*^V600E^ mutations. The RP2D is 550 mg/m2 twice daily. An efficacy cohort in patients under 25 years of age has recently completed accrual at the RP2D and will be reported separately once data matures. The upper age limit of the efficacy trial was extended to 25 years to accelerate our accrual due to the overall rarity of the disease being studied.

## MATERIALS AND METHODS

### Eligibility

The study was reviewed and approved by the Institutional Review Board of each participating institution. Verbal and written informed consent was obtained from all participants or their parents with assent obtained for the appropriately aged patients. Patients under 18 years of age who had a histologically confirmed diagnosis of a BRAF^V600E^-mutant primary brain tumor and Lansky or Karnofsky performance score ≥ 60 were eligible. Confirmation of the BRAF^V600E^ mutation was required in a CLIA-approved laboratory, either by sequencing or immunohistochemistry. Either fresh biopsy or archival tissue were allowable for genotyping. Subjects must have had disease that failed at least one prior therapy (including radiation or systemic therapy) besides surgery and had to have evidence of measurable disease on MRI. There was no restriction for number of prior therapies. Subjects must have recovered from acute side effects of prior therapies and shown evidence of adequate bone marrow function (absolute neutrophil count > 1000 cells/ul, hemoglobin > 8 gm/dl and platelets > 75,000/ul), renal function within normal limits for age and liver function (total bilirubin < 1.5 × ULN for age, ALT and AST < 2.5 × ULN for age). Corticosteroids had to be on a stable or decreasing dose prior to treatment. The QTc on pre-treatment EKG had to be < 450 msec. Patients must have been able to swallow tablets.

Children with active lesions suspicious for keratoacanthoma or cutaneous squamous cell carcinoma were excluded as were subjects with a known diagnosis of Neurofibromatosis Type 1 or any other RAS-opathy due to risk of paradoxical activation of tumor growth [18]. Further, prior exposure to a BRAF inhibitor constituted an exclusion criteria.

### Treatment regimen, administration, and dose escalation design

Vemurafenib (RO5185426; PLX4032) was supplied in 120 mg and 240 mg tablets by Genentech, Roche. Study drug was administered orally twice daily (BID) in 28-day cycles. Doses were adjusted based on body surface area (m^2^) prior to each cycle. The starting dose level (dose level 0) was 550 mg/m^2^ BID twice daily which was equivalent to the adult recommended phase 2 dose (RP2D) of 960 mg based upon an average adult BSA of 1.73 m^2^. The maximum dose administered in this trial was capped at 960 mg BID. Planned de-escalation dose levels included dose level -1 420 mg/m^2^, dose level -2 330 mg/m^2^, dose level -3 240 mg/m^2^ BID on a 28-day cycle and dose level -4 240 mg/m^2^ day 1–7 and 15–21. Dosing nomograms were used to accommodate the available pill sizes. Dose levels -1 and -2 were only considered for study subjects if the body surface area (BSA) was greater than 0.75 m^2^ and dose level -3 and -4 only when BSA was greater than 0.9 m^2^.

### Definition of dose limiting toxicity (DLT)

Toxicities were graded based on the NCI Common Terminology Criteria for Adverse Events (CTCAE) version 4.0. Dose limiting toxicities (DLT) were based on adverse events occurring in the first 28 day cycle. Hematologic DLT was defined as any treatment related grade 4 hematologic toxicity with the exception of lymphopenia and anemia, grade 3 neutropenia with fever; or grade 3 thrombocytopenia. Non-hematologic DLTs were initially defined as any grade 3 or 4 related non-hematologic toxicity. The protocol was subsequently amended to exclude grade 3 rash, diarrhea, infection, fever, or photosensitivity that resolved to grade ≤ 2 within 7 days of appropriate medical management in the DLT definition. This was based on additional Genentech/Roche trial experience supporting that these specific side effects can be well managed and should not constitute a DLT. Any related grade 2 non-hematological toxicity that persisted for more than 7 days and was considered sufficiently medically significant or sufficiently intolerable by patients to warrant treatment interruption and/or dose reduction was also considered dose-limiting.

### Definition of response

Disease evaluation was by MR imaging on 1.5T or 3T clinical scanner that occurred at baseline followed by every 2-months assessments. Standard clinical sequences included 3 plane localizer, axial T2 weighted imaging, 3D fluid attenuated inversion recovery (FLAIR), and T1 weighted imaging without and with intravenous gadolinium. Subjects had the option to switch to every 3 months assessment after cycle 24. All images were anonymized prior to a retrospective central review by a study assigned neuroradiologist. Response criteria used were a modified version of the published “RANO” criteria [15]. Given that tumor cysts are commonly found in low grade gliomas in children, these were not excluded from measurements, as noted below. Measurable disease was defined as lesions that can be accurately measured in two dimensions with a minimum size of no less than double the slice thickness. T2 FLAIR sequences were used for disease assessment. All tumor measurements were recorded in millimeters or decimal fractions of centimeters and expressed as sum of products of largest diameter and perpendicular diameter. Tumor measurements over time were performed side by side in single session to maintain corresponding plane of view. Many of the lesions had both solid and cystic components. Tumor measurements included measurement of a solid portion of the tumor in lesions where the solid portion could be measured in isolation. In tumors with multiple cystic components that were inseparable from solid component, combined measurement of the “solid/cystic” lesion was performed. In tumors with only measurable cystic component, measurement of the cyst was performed. Previously irradiated lesions were considered non-measurable except in cases of documented progression of the lesion since the completion of radiation therapy. Complete response (CR) was defined as complete disappearance of the target lesion and no new lesions; partial response (PR) was defined as > 50% tumor reduction in product of bi-dimensional tumor measurements of solid lesions. Progressive disease (PD) was defined as an increase in product of bi-dimensional tumor measurements > 25% in a solid lesion or the appearance of a new lesion. For confirmation of CR and PR, results needed to be sustained for 8 weeks.

### Pharmacokinetics

#### Pharmacokinetic sampling

Serial blood samples for PK were collected on days 1, 15 and 22 in cycle one of vemurafenib treatment. Whole blood samples were collected at 2, 4, 8, and 24 hours post-dose on day 1 with additional trough levels obtained just prior to (t = 0) and 1-hour post dose on days 15. PK collection was then repeated on day 22 with samples collected just prior to (t = 0) and 2, 4, 8, and 24 hours post-dose. Two milliliters of blood was collected at each sampling time through a venous catheter and placed in a heparinized tube for vemurafenib analysis. All samples, within 30 minutes of collection, were centrifuged at 3400 rpm for 10 minutes at 4° C, and the plasma removed and stored at –80° C until analysis.

#### Bioanalysis

Plasma samples were analyzed by Covance Inc. (Princeton, New Jersey) using a validated reverse phase high performance liquid chromatography with mass spectrometry. The assay was linear in the range of 25.0 to 50,000 ng/ml RO5185426 (vemurafenib, PLX4032). Samples with concentrations above the upper limit of linearity were diluted and re-assayed. Samples with vemurafenib levels reported below the lower limit of quantification (LLOQ) were entered into the PK analysis as half the value of the LLOQ. Assay accuracy, intra-day, and inter-day variability ranged from 90.4–105.9%, 1.0–7.3%, and 1.8–6.1%, respectively.

#### Pharmacokinetic analysis

A nonlinear mixed effects modeling approach was used to describe vemurafenib time-plasma concentration data. A recent publication from the same authors described the population pharmacokinetic analysis in detail [19]. The non-linear mixed effect modeling approach has been used for characterizing the population pharmacokinetics in the past half-century [20]. Briefly, a one-compartment model with first-order absorption and elimination was applied, with a bodyweight-based allometric component added to all clearance and volume parameters using a fixed exponent of 0.75 and 1, respectively. Between-occasion variability was included in the final covariate model using an exponential equation. The area-under-the-curve (AUC) for each patient was derived from the empirical Bayes estimate of individual clearance (CL) (AUC = Dose/CL).

### Statistical design

Due to the relative scarcity of BRAF^V600E^-mutant recurrent pediatric brain tumors and the expected difficulty in accrual for a traditional dose-escalation study, this study was conducted to determine the safety of the adult RP2D equivalent of 960 mg BID (based on the average adult size of 1.73 m^2^), namely 550 mg/m^2^/dose BID (dose level 0).

Three patients were initially enrolled at the dose level 0 and observed for toxicity during the dose-finding period (cycle 1). The dose escalation and de-escalation followed a modified 3+3 design as follows: if more than 1 DLT occurred in the first 3 patients at dose level 0, that dose was declared intolerable and subsequent patients were enrolled into the lower dose cohorts described earlier. If, on the other hand, 1 or fewer DLTs were observed at dose level 0, 3 additional patients were enrolled into that dose. Barring excessive toxicity, accrual to any dose level would be complete once 6 patients had been treated, and no more than 1 patient in a cohort of 6 experienced a DLT. There were 4 lower dose levels allowed, and dose escalation beyond dose level 0 was not allowed.
